# Whole-body diffusion-weighted imaging for staging lymphoma: are apparent diffusion coefficient derived histogram parameters useful for lesion characterisation?

**DOI:** 10.1186/1470-7330-14-S1-S9

**Published:** 2014-10-09

**Authors:** K De Paepe, F De Keyzer, P Wolter, O Bechter, A Janssens, D Dierickx, R Oyen, G Verhoef, V Vandecaveye

**Affiliations:** 1 University Hospitals Leuven, Leuven, Belgium

## Aim

To evaluate apparent diffusion coefficient (ADC) derived histogram parameters for lesion characterization in whole-body diffusion-weighted imaging (WB-DWI) of lymphoma.

## Methods

Fifteen patients with histopathology proven lymphoma (11 Non-Hodgkin; 4 Hodgkin lymphomas) underwent WB-DWI using 2 b-values (0-1000 s/mm^2^). On coronal reformatted b1000 WB-DWI images, regions of interest (ROI) were drawn semi-automatically on lymph nodes in all nodal stations (n=267) and in axial and appendicular bone regions (n=53). For each ROI, a histogram was constructed from which volume, mean_(ADC)_, median_(ADC)_, skewness_(ADC)_, and kurtosis_(ADC)_ were calculated. Mann-Whitney-U tests were performed to detect significant differences between malignant and benign ROIs per tissue type. Receiver-operating-characteristic curves (ROC) were constructed from which an optimal threshold was determined as well as sensitivity, specificity and accuracy. PET/CT plus bone marrow biopsy (BMB) served as reference standard.

## Results

All parameters were significantly different between malignant and benign lymph nodes (p<0.001) with skewness_(ADC)_ being the most accurate. A positive skewness exceeding 0.3041 mm^2^/s allowed for detection of malignant lymph nodes with 88% accuracy, 88% sensitivity and 87% specificity compared to 63% accuracy, 61% sensitivity and 64% specificity for mean_(ADC)_. Only kurtosis_(ADC)_ (p<0.001) and skewness_(ADC)_ (p=0.003) were significantly different between malignant bone marrow infiltration and normal bone marrow. Kurtosis_(ADC)_ showed highest accuracy and a threshold exceeding 5.26 allowed for detection of malignant bone marrow infiltration with 89% accuracy, 86% sensitivity and 90% specificity.

## Conclusions

ADC histogram analysis is feasible for lesion characterization in WB-DWI of lymphoma. Lymph nodes were most accurately characterized using skewness_(ADC)_ and bone tissue using kurtosis_(ADC)_.

